# Role of YAP as a Mechanosensing Molecule in Stem Cells and Stem Cell-Derived Hematopoietic Cells

**DOI:** 10.3390/ijms232314634

**Published:** 2022-11-23

**Authors:** Nattaya Damkham, Surapol Issaragrisil, Chanchao Lorthongpanich

**Affiliations:** 1 Siriraj Center of Excellence for Stem cell Research, Faculty of Medicine Siriraj Hospital, Mahidol University, Bangkok 10700, Thailand; 2 Division of Hematology, Department of Medicine, Faculty of Medicine Siriraj Hospital, Mahidol University, Bangkok 10700, Thailand; 3 Bangkok Hematology Center, Wattanosoth Hospital, BDMS Center of Excellence for Cancer, Bangkok 10310, Thailand

**Keywords:** YAP, stem cells, differentiation, hematopoietic stem cells, mechanical forces, mechanosensing

## Abstract

Yes-associated protein (YAP) and WW domain-containing transcription regulator protein 1 (WWTR1, also known as TAZ) are transcriptional coactivators in the Hippo signaling pathway. Both are well-known regulators of cell proliferation and organ size control, and they have significant roles in promoting cell proliferation and differentiation. The roles of YAP and TAZ in stem cell pluripotency and differentiation have been extensively studied. However, the upstream mediators of YAP and TAZ are not well understood. Recently, a novel role of YAP in mechanosensing and mechanotransduction has been reported. The present review updates information on the regulation of YAP by mechanical cues such as extracellular matrix stiffness, fluid shear stress, and actin cytoskeleton tension in stem cell behaviors and differentiation. The review explores mesenchymal stem cell fate decisions, pluripotent stem cells (PSCs), self-renewal, pluripotency, and differentiation to blood products. Understanding how cells sense their microenvironment or niche and mimic those microenvironments in vitro could improve the efficiency of producing stem cell products and the efficacy of the products.

## 1. Introduction

Accumulating evidence strongly suggests that cell biological changes are regulated not only by internal soluble factors (cytokines and hormones) but also by physical and mechanical cues. The type of extracellular matrix (ECM) stiffness and adhesiveness are mechanical stimuli currently being studied [1,2,3]. Through mechanotransduction systems, cells translate these stimuli into biochemical signals that regulate multiple aspects of cell changes, such as growth and differentiation. How mechanical impulses are linked to the activity of nuclear transcription factors remains poorly understood.

**Figure 1:**
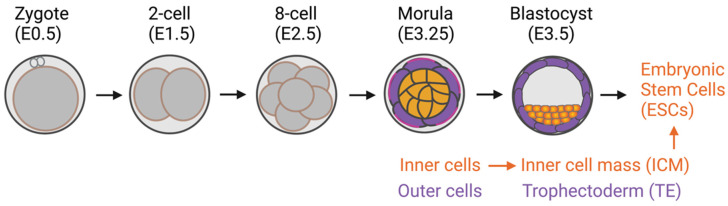
Preimplantation mouse embryo development. Inner cells with high adhesive forces acquire their inner cell mass fate, a source of embryonic stem cells. The outer cells have lower adhesive forces and become trophectoderm cells.

In addition to the classical role of the Hippo signaling pathway in regulating cell proliferation and apoptosis [4,5,6], the pathway has been demonstrated to be one of the mechanosensing pathways that convey the mechanical signals that modulate cell function. In the mammalian preimplantation embryo, positional sensing ability is crucial for the trophectoderm (TE)-inner cell mass (ICM) fate decision. Through adhesiveness, each embryonic blastomere can sense its positioning within an intact preimplantation embryo. The blastomeres receiving high adhesiveness, i.e., those in the inner cell, can secure their inner cell mass fate, the origin of embryonic stem cells (ESCs). However, the blastomeres in the outer layer of the embryo, i.e., the outer cells, receive fewer adhesive stimuli and become trophectoderm cells [7,8,9] (Figure 1). It has previously been shown that the Hippo component proteins (large tumor suppressor kinases 1/2 (LATS1/2), mammalian STE20-like protein kinase 1/2 (MST1/2), and YAP) are responsible for translating positional information to lineage specification through the cell adhesiveness positional-sensing mechanism [10,11]. Disruption of the Hippo pathway-component gene in early embryos leads to failure of lineage specification and postimplantation development due to the loss of positional sensing information [10,11,12]. In addition to the mammalian preimplantation embryo, the role of the Hippo pathway in mechanotransduction has been implicated in other cells: cancers, mesenchymal stem cells (MSCs), and endothelial cells [13,14].

As cited above, there have been reports on the role of Hippo-YAP/TAZ pathway regulation and its biological roles. This review focuses on a novel role of YAP, an effector protein of the Hippo pathway, and its homolog TAZ as mechanosensing molecules altering the mammalian stem cell pluripotency state and differentiation capacity. The review also examines the impact of YAP/TAZ on human hematopoiesis, especially during the formation of PSC-derived blood products, including hematopoietic stem cells (HSCs), T cells, megakaryocytes, and red blood cell differentiation.

### 1.1. The Hippo-YAP/TAZ Signaling Pathway

The Hippo signaling pathway was first identified in *Drosophila melanogaster* through genetic screening [15,16]. Later studies revealed its conserved role in regulating organ size, cell fate, cell growth, and apoptosis in other mammals, including humans [17,18,19,20]. YAP is a critical transcriptional coactivator and a crucial effector protein that regulates downstream target genes involved in cell proliferation and differentiation, namely, *Cyclin A*, *Myc*, *Ctgf*, *Cdx2*, and *Ajuba* [5,15,16,18,19,20,21,22,23,24,25,26]. TAZ, a YAP homolog, is another well-recognized Hippo effector protein. However, its role in regulating cell function and whether its function is redundant to YAP is not well understood [27]. Since the known functions of YAP and TAZ are mainly redundant, this review used “YAP/TAZ” to refer to a YAP and TAZ protein complex unless otherwise stated.

In the classical model of the Hippo pathway, YAP/TAZ activity is negatively regulated by Hippo-LATS1/2 core kinases. In the nonactive state of the core kinases, most YAP/TAZ molecules are active and translocate into the nucleus, binding to its transcription factors (TEADs) and driving the target gene expression of the YAP/TAZ-TEAD complex [28,29,30]. Once the core kinases are activated through upstream signals such as cell-cell contacts, the activated core kinases phosphorylate YAP/TAZ, resulting in cytoplasmic retention and inhibition of downstream target gene expression [19,31,32,33,34]. However, whether the response of YAP/TAZ to mechanical stimuli depends on the canonical Hippo-LATS1/2 core kinases has yet to be delineated.

### 1.2. Stem Cells

Stem cells are cells with the ability to self-renew and differentiate into many cell types in the body [35]. Therefore, stem cells are a holy grail for regenerative medicine [36]. They are classified into four groups by their derivation source: (1) adult stem cells [36], (2) perinatal stem cells [37], (3) ESCs [38], and (4) induced pluripotent stem cells (iPSCs) [39].

Adult stem cells are undifferentiated cells that reside in tissues or organs in the adult body. The primary roles of these cells are to maintain and repair the tissue in which they reside through their self-renewal and differentiation capacity. However, adult stem cells are multipotent or unipotent, meaning they can be differentiated into distinct, but not all, cell types, depending on their tissue of origin. One of the very well-studied adult stem cell types, which will be often mentioned in this review, is MSCs. MSCs are multipotent stem cells that are found in several tissues and can differentiate into at least 3 distinct cell types: osteoblasts, adipocytes, and chondrocytes [40,41]. Gradually increasing information shows the transdifferentiation capacity of MSCs to other cell types, such as neuron-like cells [42,43,44], smooth muscle cells [45,46], and cardiomyocytes [47]. These findings support the wide clinical applications and regenerative capacity of MSCs.

Another well-known, clinically approved adult stem cell type for therapeutic approaches is HSCs. HSCs are responsible for all blood cell production through the process termed hematopoiesis. The classical model of their differentiation hierarchy is that HSCs differentiate into multipotent progenitors (MMPs) that no longer have a self-renewal ability. MPPs differentiate into common lymphoid progenitors (CLPs) and common myeloid progenitors (CMPs). In turn, CMPs differentiate into megakaryocyte–erythroid progenitors (MEPs) and granulocyte–macrophage progenitors (GMPs). Both of these progenitors then differentiate into mature cell types, including red blood cells (erythrocytes), megakaryocytes, myeloid cells (monocytes, macrophages, and granulocytes), mass cells, T- and B-lymphocytes, and natural killer cells [48,49,50,51,52,53]. However, several new hematopoietic hierarchy models have recently been proposed [54]. One is an early split model, in which the HSC lineage separates earlier than in the classical model [55,56,57,58]. Another newly described model is a continuous, Waddington-like model [54,59,60,61]. This model suggests that HSCs do not pass through a stable or discrete intermediate form but instead continuously acquire lineage-committed transcription [54,59,60,61].

Perinatal stem cells are stem cells that can be isolated from tissues that are discarded after birth, such as the placenta, umbilical cord, cord blood, and amniotic fluid. Different types of stem and progenitor cells can be isolated from these tissues. The most well-known perinatal stem cells are HSCs isolated from umbilical cord blood and MSCs isolated from umbilical cord blood and perinatal tissues, such as placenta or chorionic tissue [37]. Perinatal stem cells represent an intermediate cell type that combines the qualities of adult stem cells and ESCs and holds broad, multipotent plasticity.

Unlike adult and perinatal stem cells with limited multipotent differentiation capacity, PSCs (ESCs and iPSCs) can self-renew and differentiate into all cell types in the body, including blood cells [62]. ESCs are derived from the inner cell mass of an embryo [63]. Consequently, the related ethical issues are the most challenging aspect of their use. Yamanaka and colleagues successfully generated PSCs by reprogramming the skin fibroblasts to a pluripotent state, called iPSCs [39]. Since then, iPSCs have become the great hope of cell origin to generate personalized cells for regenerative medicine [64,65]. However, the current challenges in generating iPSC-derived target cells are their production efficiency and efficacy [66]. Further research into creating a suitable in vitro niche microenvironment to mimic an in vivo microenvironment could be one way to achieve success [67].

## 2. Mechanosensing and Forces Regulating YAP/TAZ

“Mechanosensing” is the term used to describe cells’ ability to sense mechanical cues in their microenvironment. “Mechanotransduction” refers to the ability of cells to subsequently translate and respond to mechanical cues by programming cell behaviors [68]. Many mechanical cues modulate the growth and lineage decisions of cells, including ECM stiffness, blood flow, wall or turbulent shear stress, cell shape (geometry), cell density, topographic surfaces, and cytoskeleton tension. However, how cells respond to such cues to generate biological responses is poorly understood. The first evidence of a novel function of YAP as a mechanosensing protein came from the study by Dupont and colleagues on MSCs in 2011 [13]. Their work showed that mechanical forces or cues (ECM stiffness, cell spreading, and cytoskeleton tension) mediate YAP localization and result in the lineage differentiation bias of MSCs [13]. Their study results shed light on the noncanonical role of YAP/TAZ as a mechanosensing molecule in stem cells. Since then, several models have confirmed that YAP can act as a mechanosensor to convey signals that control cell function and biological responses [69,70,71,72,73].

### 2.1. ECM Stiffness Influences MSC Differentiation via YAP/TAZ

The adipo-osteogenic balance mechanism regulates the ability of MSCs to differentiate into adipocytes or osteoblasts. Dysregulation of this balance has been linked to particular pathophysiological processes: bone loss and obesity. YAP has been reported as a central regulator controlling the balance, given that high YAP expression induces MSCs to differentiate into osteoblasts, whereas low YAP expression induces adipogenesis [74]. Uncovering the relationship between ECM matrix stiffness and YAP/TAZ has led to extensive investigations to determine whether YAP/TAZ acts as a mechanosensing molecule in response to ECM stiffness to control MSC fate differentiation into either osteoblasts or adipocytes.

**Figure 2:**
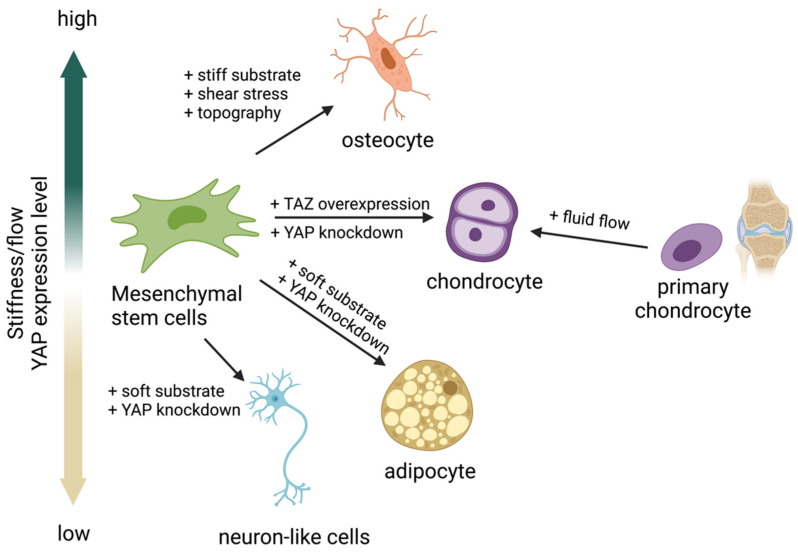
Yes-associated protein (YAP) acts as a mechanosensing molecule in mesenchymal stem cells (MSCs) fate determination.

Many studies have reported that a stiff substrate activates YAP activity, resulting in YAP/TAZ translocation into the nucleus and inducing MSC differentiation into osteoblasts [13,69,75]. In contrast, a soft substrate was reported to inhibit YAP/TAZ activity by restraining YAP/TAZ in the cytoplasm, resulting in MSC differentiation into adipocytes (Figure 2) [13,69,75,76,77]. These results suggest that the activity of YAP is crucial for MSCs to regulate the adipo-osteogenic differentiation balance while undergoing differentiation. In addition, YAP seems to play a role as a negative regulator of MSC differentiation to chondrocytes [78,79,80], while overexpression of TAZ promotes chondrocyte differentiation from MSCs [81]. In contrast, fluid shear stress promotes chondrocyte maturation from the primary chondrocyte progenitor [82]. These findings suggest that both YAP/TAZ and fluid shear stress regulate chondrocyte differentiation. Modulating YAP activity using matrix stiffness or fluid shear stress could direct differentiation into the desired cell type without genetic alternation. This approach could be applied to the production of adipocytes, osteoblasts, or chondrocytes for clinical use, and it may facilitate tissue regeneration [13,69,75,76,77,83].

Several research groups now focus on the transdifferentiation ability of MSCs to cell types other than adipocytes, osteoblasts, and chondrocytes. There have been attempts to differentiate MSCs into neurons [42], corneal epithelial cells [84], keratinocytes [85], and several other cell types. However, success in obtaining fully differentiated cells has been limited. Applying knowledge of creating a biological microenvironment to mimic the in vivo niche and applying a suitable ECM type and stiffness are likely to enhance the degree of differentiation.

### 2.2. Fluid Shear Stress and Force Modulate YAP/TAZ Activity

**Figure 3:**
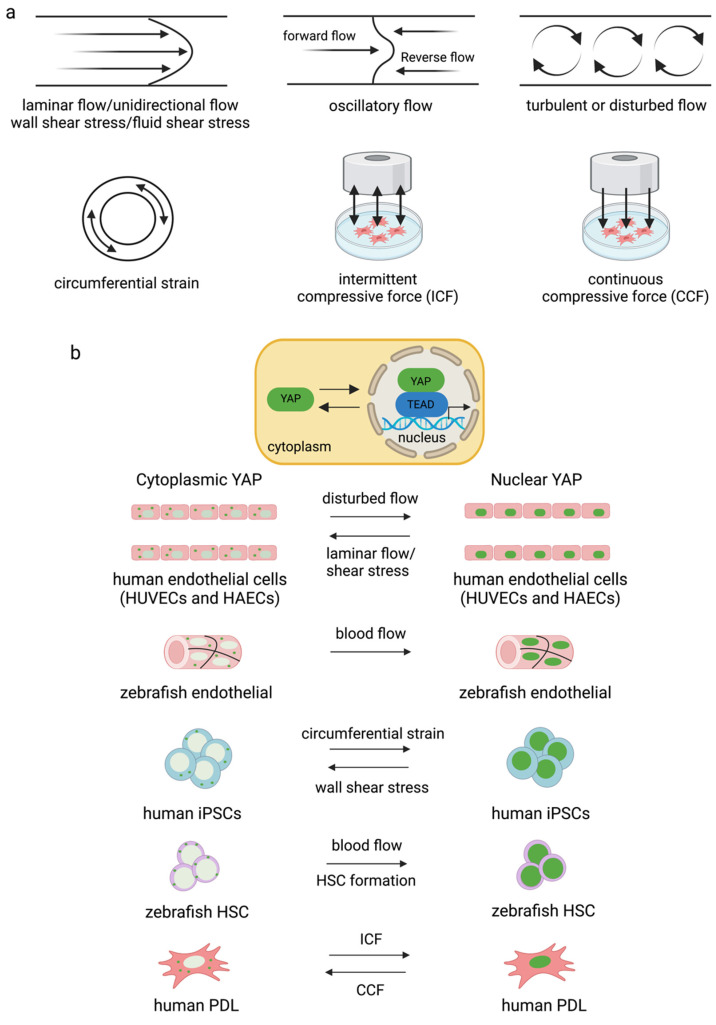
Different types of flow and strain mediate YAP/TAZ activity in different cell types (**a**,**b**). Disturbed flow increased YAP activity in endothelial cells [86,89] and blood flow induced nuclear YAP in zebrafish vessels [87]. Circumferential strain induced YAP expression in human iPSCs, and blood flow induced YAP translocated into the nucleus for HSC formation in zebrafish [88]. ICF and CCF mediated YAP expression differently in human PDL [83].

Fluid shear stress and force have been found to modulate YAP expression. Several forms can modulate YAP/TAZ activity. They are laminar flow [86,87], disturbed or oscillatory flow [86], circumferential strain [88], fluid shear stress [82], wall shear stress [88], intermittent compressive force (ICF), and continuous compressive force (CCF) [83] (Figure 3). Interestingly, different flow patterns activated YAP nuclear activity to various degrees. It has been shown that applying shear stress to epithelial cells to mimic blood flow induced YAP activity by enhancing nuclear localization in zebrafish endothelial cells [87]. Disturbed flow induced nuclear YAP, while laminar flow or shear stress inhibited YAP in human endothelial cells [86] and human iPSCs [88]. Circumferential strain promoted YAP expression in human iPSCs [88]. ICF increased YAP expression, while CCF reduced YAP expression in the human periodontal ligament and MSC-like cells isolated from tooth connective tissue [83]. There are limited reports on the effects of fluid shear stress on YAP/TAZ activity and cell biological changes relative to the number of studies investigating how ECM works. Further experiments are needed to improve our understanding of the effects of the bloodstream on the differentiation capacity and function of blood cells.

## 3. YAP Regulates the Self-Renewal, Pluripotency, and Differentiation of PSCs, Dependent on ECM Stiffness and Cytoskeleton Tension

In a monolayer culture of epithelial cells, it is known that cell-contact signals inhibit YAP translocation into the nucleus. This effect is termed the “cell-contact inhibition” mechanism [19]. Cell contact inhibition refers to the cell growth arrest that occurs when cells come into contact with each other. As a result, cell proliferation is inhibited while triggering apoptosis. Contact inhibition is a potent anticancer mechanism that is lost in cancer cells [90].

In contrast, the cell-contact inhibition mechanism cannot be applied to PSCs, as the cells are tightly packed with a high degree of cell–cell contact signaling [90]. Nevertheless, YAP is still active and highly expressed in the nucleus of undifferentiated PSCs [70]. Consequently, it may be that cell-contact inhibition signaling cannot activate Hippo core kinases in PSCs. Therefore, the Hippo-YAP signaling pathway in PSCs must be regulated differently from other cell types, including MSCs [91].

### 3.1. Role of YAP in Stem Cell Pluripotency, Self-Renewal, and Differentiation

YAP is active and highly expressed in PSCs and is mainly localized in the nucleus of undifferentiated PSCs. Downregulation of YAP in mouse ESCs leads to a loss of ESC pluripotency and induces differentiation. Conversely, YAP overexpression inhibits mouse ESCs from differentiating even under differentiation conditions [92]. Mechanistically, Tamm et al. (2011) [93] showed that, in the presence of leukemia inhibitory factor and IαI factor in serum, YAP binds to the transcription factor TEAD. Consequently, OCT3/4 and NANOG expression occurs, and the pluripotent state of mouse ESCs is maintained [94]. YAP has since been recognized as a crucial factor playing a significant role in the regulation of self-renewal and the pluripotency of mouse ESCs [32]. However, it must be noted that the mechanism for maintaining pluripotency and self-renewal of human ESCs is distinct from that of mice. Although basic fibroblast factor (bFGF) is crucial for humans, leukemia inhibitory factor signaling is critical for mouse PSCs. Therefore, mouse findings may not directly apply to humans [91].

Several studies have demonstrated that YAP and TAZ are highly expressed in human ESCs and iPSCs [70,95,96,97,98]. YAP overexpression facilitates the reprogramming efficiency of human amniotic epithelial cells into iPSCs [99]. However, the role of YAP/TAZ in maintaining pluripotency, self-renewal, and differentiation remains controversial [98,100,101]. In contrast to mice, recent studies reported that YAP is indispensable for human PSCs to maintain their pluripotency state [100]. Lorthongpanich and colleagues further demonstrated that depletion of YAP or TAZ did not alter the pluripotency, self-renewal, or teratoma formation of human iPSCs [101,102]. A growing body of evidence suggests that the reduction of YAP/TAZ activity has no significant effect on the stemness of human PSCs. Nevertheless, the role of YAP/TAZ in differentiating PSCs into different cell types remains to be determined.

Human pluripotent stem cells are classified into primed and naïve states based on their growth characteristics and potential to differentiate into all three embryonic lineages and the germ line in chimeras [103]. Human-ESCs and iPSCs identified more closely with the primed state [100]. Upon specific induction, the primed state PSCs can be reverted to the naïve state. This reversion could improve the differentiation efficiency and enhance the generation of gene-corrected autologous pluripotent stem cells. Understanding the requirements for induction and maintenance of the naïve pluripotent state will significantly drive the application of stem cells for therapeutic usage.

There have been attempts to revert primed state hESCs and iPSCs to the naïve state by the forced expression of transcription factors, i.e., OCT4, SOX2, NANOG, KLF4, and KLF2 [103]. However, one limitation of this approach is that continuous expression of some transcription factors, such as OCT4, NANOG, and SOX2, restricts the differentiation of pluripotent stem cells to specific cell types. To overcome this limitation, several groups have used small molecules to achieve transgene-independent derivation of naïve hPSCs. To support the growth of naïve human PSCs, various combinations of small molecules and growth factors have been used, such as 2i/LIF, TGFβ1, c-Jun N-terminal kinase inhibitor (JNKi, SP600125), p38i (SB203580), Rho kinase inhibitor (ROCKi, Y-27632), and protein kinase C inhibitor (PKCi, Go6983) [104]. In 2016, Qin and colleagues found that activating YAP activity by supplementing lysophosphatidic acid (LPA) in mTeSR+2iFL media significantly induced the transition from the primed to the naïve state in multiple human ESC and iPSC lines, and the naïve state was prolonged in the culture medium supplemented with LPA [100]. These results suggest an unexpected role of YAP in regulating the induction and maintenance of human naïve stem cells.

### 3.2. ECM Stiffness and Cytoskeleton Tension Influence YAP Activity-Mediated Stem Cell Differentiation

The upstream regulators of YAP related to mechanical forces in PSCs, including substrate stiffness [105] and surface topography [106], have been studied recently. The role of mechanical forces in embryogenesis, ECM, and topology (surface) design to support human ESC self-renewal and pluripotency has been demonstrated [107,108,109]. It has been shown that the stiffness of the coating material modulates YAP activity in PSCs. A soft substrate (a hydrogel) inhibits YAP activity and delays stem cell proliferation; however, a stiff substrate supports cell proliferation better than the softer, synthetic hydrogel substrate [105,110]. These findings suggest that stiff material enhances YAP activity and that higher YAP levels in the nucleus support the long-term self-renewal of human ESCs.

Other than external factors such as ECM type and stiffness, YAP/TAZ activity in human PSCs can also be mediated by internal factors, specifically, the organization of the actin cytoskeleton at the cell membrane and Rho activity [98]. The levels of polymerized filamentous (F)-actin have a positive association with cell attachment and cell spreading morphologies. Detached cells have lower levels of F-actin than adhered cells [105]. Moreover, the Rho/F-actin cascade supports human ESC survival and expansion by promoting YAP/TAZ nuclear localization and YAP/TAZ activity [98]. Overall, these data suggest that there are orchestrating functions of external (ECM stiffness) and internal factors (cytoskeleton tension) that regulate stem cell differentiation through the activation of YAP.

**Figure 4:**
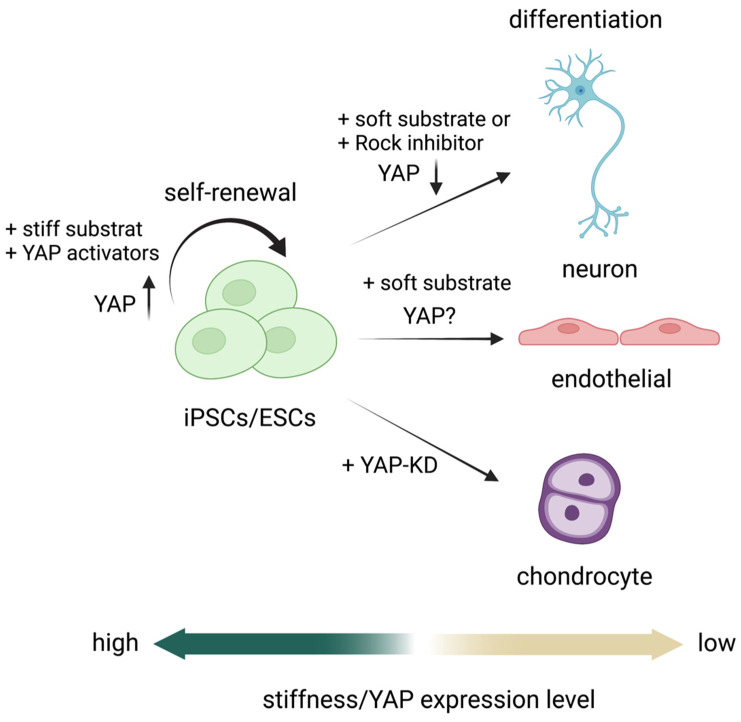
Role of YAP as a mechanosensor in PSC self-renewal [97] and differentiation into neurons [70,97,111,114], endothelial cells [114], and chondrocytes [112].

The role of nuclear YAP activity in regulating the pluripotency and self-renewal of PSCs remains controversial. However, it is widely accepted that low nuclear YAP activity promotes stem cell differentiation. The stiffness of the coating substrate modulates YAP activity in PSCs. It has been shown that culturing iPSCs on a soft or compliant substrate inhibits YAP activity but promotes iPSC differentiation into neuron progenitor cells and postmitotic neurons. This corresponds to YAP activity in that a stiff substrate enhances YAP activity, whereas a soft substrate reduces it [42]. Additionally, adding small molecules, such as the ROCK inhibitor Y-27632, inhibits YAP/TAZ activity by promoting its cytoplasmic localization, significantly increasing the percentage of differentiated neuroepithelial progenitors. In contrast, adding LPA to promote YAP/TAZ nuclear localization inhibits neuron induction [111]. Other than neurons, a reduction of YAP activity by modulating ECM stiffness has been demonstrated to promote the differentiation of PSCs into chondrocytes and endothelial cells [112,113]. The comprehensive activity of YAP and stiffness in PSC differentiation into some specific cell types are illustrated in Figure 4.

### 3.3. Small Molecules Targeting YAP/TAZ Activity via the Actin Cytoskeleton

Several small molecules and agents can modulate YAP/TAZ activity. The inhibition can directly target the Hippo pathway component proteins or indirectly target the actin cytoskeleton using inhibitors such as ROCK.

Cytochalasin D, an actin inhibitor, blocks actin polymerization, resulting in YAP and TEAD nuclear activity reduction. Cytochalasin D was used to study the role of actin cytoskeleton-mediated YAP activity in mouse embryonic fibroblasts and HEK293 cells [115,116]. C3 exoenzyme, a Rho-specific inhibitor, was used in human ESCs [98] and human endothelial cells [86] to study the role of Rho. Latrunculin A and B were used to study the role of actin polymerization-mediated YAP sensing in several cell types [13,70,115,116,117]. Summaries of the small molecules that target YAP/TAZ and those that mediate YAP/TAZ activity via the cytoskeleton are presented in Table 1 and Table 2, respectively.

## 4. Role of YAP during HSC Formation and Blood Cell Production

### 4.1. Role of YAP during HSC Formation

As mentioned earlier, PSCs can differentiate into all types of cells in the body, including blood cells, via in vitro hematopoiesis. PSC-derived HSCs are one of the most desired blood products, as HSCs are potent starting cells that can be further differentiated into all blood cell types. However, there are still challenges to be overcome regarding production efficiency. The Hippo pathway has been linked to hematopoiesis since the novel role of the pathway in regulating blood cell production was first demonstrated in *Drosophila* in 2014 [124,125,126]. The pathway was later implicated in mammalian hematopoiesis [95].

Bioinformatic gene regulatory network analysis of mouse ESC differentiation into HSCs and macrophages revealed that YAP/TEAD binds to Tal1 and Fli1 transcription factors during hemangioblast transition to hemogenic endothelial cells [127]. YAP/TEAD is also involved in hematopoietic specification and differentiation in the hemogenic–endothelial transition stage during mESC differentiation into macrophages in vitro [127]. In addition, YAP/TAZ has recently been demonstrated as an essential molecule to regulate HSC fitness, self-renewal, and differentiation fate through interaction with the Scribble protein. The combined loss of Scribble, YAP, and TAZ results in transcriptional upregulation genes involved in HSC fitness in mice [128]. Studies on zebrafish and human iPSC-derived HSCs further confirmed the role of YAP/TAZ in HSC formation [88]. However, YAP seems dispensable for normal and malignant hematopoiesis in mice [129,130]. Recently, the upstream mediators of Lats1/2 and YAP, MST1/2, have been reported to be indispensable molecules in HSC formation. Deleting MST1/2 markedly altered the maturation of HSCs and HSC-derived blood cells [131]. Overall, it can be concluded that the Hippo pathway contributes substantially to HSC production and fate.

### 4.2. Role of YAP in Myeloid and Lymphoid Lineage Development

#### 4.2.1. Role of YAP in T-Cell Development and Activation

The roles of YAP and TAZ have been determined in Treg and T helper 17 (TH17) cell fate differentiation [132,133]. YAP is required for the generation and function of Treg [132], while TAZ has been shown to promote TH17 cell differentiation from naïve CD4^+^ T cells [133]. It was demonstrated that the sensing of stiffness by YAP had a critical role in a mouse model during T-cell activation after viral infection. It has been reported that node stiffness increased by approximately 10-fold due to lymphoproliferation. This increased stiffness activated the YAP in T cells, resulting in T-cell activation. Similarly, YAP expression and T-cell activation were elevated when cultured on stiff hydrogels mimicking lymph node stiffness. The YAP sensing of lymph node stiffness appears to mediate the feedback mechanism of T cells during viral infections [134].

#### 4.2.2. Role of YAP in Megakaryocyte Differentiation and Platelet Production

The role of YAP/TAZ in human megakaryocyte differentiation was determined using the MEG-01 cell line and cord-blood-derived megakaryocytes/platelets as a model [95,135]. LATS and YAP have an essential role in megakaryoblast proliferation, maturation, and platelet production, whereas TAZ showed a minor effect [135]. Increasing YAP activity induced megakaryocytic cell proliferation but inhibited maturation, resulting in low platelet production. Conversely, YAP reduction inhibited proliferation but increased platelet production [95]. These results suggest that the dynamic expression of YAP during megakaryopoiesis is essential for megakaryocytic cell growth. Modulating YAP activity using small molecules may present an opportunity to achieve the large-scale in vitro production of platelets for transfusion.

#### 4.2.3. Role of YAP in Red Blood Cell Maturation and Enucleation

The role of YAP in mouse blood cell production has been studied using transgenic mice as a model. YAP1 knockout in mice was created by having YAP deleted in all HSCs, a starting cell in the blood differentiation lineage. Consequently, YAP was deleted from all the subsequent HSC-derived blood cells. However, the results showed that the absence of YAP had no significant effects on overall blood cell production (myeloid, lymphoid, and red blood cells) but showed a minor effect on the anemia phenotype [130]. The overexpression of YAP in hematopoietic cells also did not alter normal hematopoietic stem cell function in mice [129]. However, under stress conditions, YAP was crucial for promoting erythroid progenitor proliferation in mice [136].

Recently, we demonstrated that both YAP and TAZ are essential for human erythroid differentiation and maturation from HSCs isolated from umbilical cord blood and mobilized peripheral blood. Depleting either YAP or TAZ during human erythroid differentiation from HSCs significantly impaired erythroblast maturation and resulted in the inhibition of the enucleation of erythrocytes. It is suggested that YAP and TAZ are required in the late stage of human erythropoiesis. However, the transient overexpression of YAP or TAZ in erythroblasts does not have any apparent effect on erythroid maturation and enucleation [122].

### 4.3. ECM Stiffness and Mechanical Force Affect YAP Activity during HSC Differentiation

Evidence supports the contention that YAP acts as a mechanosensor during HSC formation. Studies were conducted on the effects of circumferential strain and wall shear stress on HSC formation using human PSCs and on the effects of blood flow in zebrafish. The research demonstrated that circumferential strain significantly upregulated the expression of YAP and its target genes (*ANKRD1* and *CTGF*) [88]. Moreover, circumferential strain increased the total number of colony-forming units, whereas wall shear stress downregulated the target genes compared with the static control.

Mechanistically, blood flow stimulates YAP activity via a Rho-GTPase-mediated mechanism in the ventral dorsal aorta. Conversely, the loss of YAP activity in zebrafish led to reduced expression of the HSC markers *Runx1* and *Cmyb*. These results demonstrated that YAP is required and mediates mechanosensing during developmental hematopoiesis in vivo (zebrafish) and in vitro (human PSCs) [88]. The results also correspond to prior studies, which found that different flow patterns induced YAP activity differently in endothelial cells [86,87] and plasma membranes [137,138].

**Figure 5:**
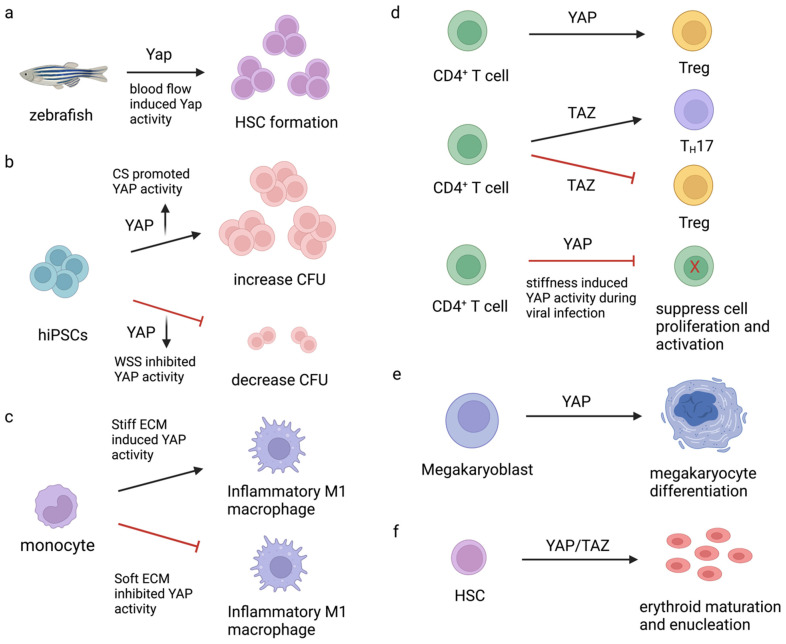
Role of YAP/TAZ in HSC formation and blood cell development. (**a**) Blood flow-induced YAP activity promoted HSC formation in zebrafish embryos [88]. (**b**) Different flow types; CS (circumferential stretch) vs. WSS (wall shear stress)-induced YAP activity differently to control colony-forming unit (CFU) formation in hiPSCs [88]. (**c**) ECM stiffness mediated-YAP activity in macrophage inflammatory responses [139]. (**d**) Role of YAP and TAZ in CD4^+^ T cell differentiation and functions [132,133,134]. (**e**) Role of YAP in megakaryocyte differentiation [95]. (**f**) YAP plays a critical role during erythrocyte maturation and enucleation from HSC [122]. Forward arrows define the promotion or requirement; blunt arrows refer to the inhibition. Upward arrows and downward arrows define the up-regulation and down-regulation, respectively.

ECM stiffness has been found to mediate YAP activity during monocyte-derived macrophages. Adhesion to soft substrates reduced TNF-α pro-inflammatory responses, whereas adhesion to stiff materials increased its responses. Regulation of YAP by ECM might help regulate macrophages in human health and diseases [139]. A summary of the roles of YAP and TAZ in HSC formation and blood cell development is illustrated in Figure 5. The modulation of YAP/TAZ by mechanical forces may be used to produce the desired cell types, e.g., red blood cells, platelets, or immune cells, which can be used for cell transplantation and blood transfusions in the future.

### 4.4. Effect of Blood Flow on YAP in Endothelial Cell Maintenance, Diseases, and Anti-Inflammatory Effects

Endothelial cell proliferation, migration, and remodeling regulate blood vessel formation and development. YAP and TAZ have been reported to play a role in endothelial progenitor cells. Depleting both YAP and TAZ decreased endothelial progenitor cell survival and impaired the critical functions of the cells (migration, invasion, vessel formation, and expression of proangiogenic genes) [140].

In endothelial vessels, endothelial cells directly contact and sense blood flow. An abnormality in the flow, such as turbulence, might give rise to atheroprone endothelial phenotypes and atherosclerosis. Studies on YAP’s response to the blood flow in endothelial cells and disturbances to the flow revealed that YAP/TAZ could sense different flow types and generate different responses. Importantly, activation of YAP/TAZ by disturbed flow promoted atheroprone endothelial phenotypes and atherosclerosis [86,89].

Similar results were found in Nakajima and colleagues’ study on the effects of blood flow in the endothelial cells of zebrafish and humans. They found that, in vitro, laminar shear stress (designed to mimic blood flow) induced translocation of YAP to the nucleus of human pulmonary artery endothelial cells within 10 min. The translocation decreased after applying the flow for 6 h. The study findings imply that hyperactivation of YAP can trigger the homeostatic balance mechanism in blood vessels. Likewise, in an in vivo study, blood flow also induced translocation of YAP to the nucleus of vascular endothelial cells of zebrafish [87]. These findings indicate that YAP is regulated by blood flow in endothelial cells. Turbulent flow induced nuclear translocation of YAP, while a unidirectional laminar flow induced YAP nuclear localization transiently and then reduced YAP nuclear activity by promoting YAP cytoplasmic retention and degradation [86,87,89]. In addition, YAP1 mutant zebrafish showed a defect in vascular stability, indicating that YAP is required for vessel maintenance [87].

Overall, the results suggest that YAP/TAZ is a crucial mechanosensing molecule in endothelial blood vessels. The activity of YAP/TAZ can be regulated by blood flow. Endothelial cells with hyperactivity of YAP/TAZ develop vessel inflammation, resulting in organ and tissue damage. The selective targeting of YAP in endothelial vessels might be a therapeutic approach to prevent or cure atherosclerosis in the future.

## 5. Concluding Remarks and Perspectives

YAP and TAZ, transcriptional coactivators of the Hippo pathway, have been discovered to have an original function in cell proliferation and tissue growth. YAP is becoming an attractive effector protein since it is critical in promoting cell proliferation in normal and cancer cells. Moreover, YAP/TAZ plays a significant role in stem cell differentiation. YAP is one of the most well-known critical players in mechanosensing and mechanotransduction. YAP senses and translates mechanical forces from the microenvironment and regulates downstream responses to program stem cell differentiation. However, how YAP/TAZ senses those mechanical cues are largely unknown. There are reports demonstrating that YAP/TAZ senses those mechanical cues by specific biological ligands [110], integrins [89], or plasma membrane caveolae, caveolin, and cavin-1 [137,138]. Understanding how mechanical forces control YAP/TAZ localization could enable the development of culture conditions that promote cell differentiation into specific lineages and increase the production yields of target cells by a transgene-independent approach.

## Figures and Tables

**Table 1:** Small-molecule-mediated YAP/TAZ activity.

Agents	Effectiveness	Cell Types	Treatment Results	References
Dasatinib	Chemotherapeutic agent	Human MSC-derived chondrocyte	YAP and TAZ inhibitors increased YAP phosphorylation at serine 127	[118]
Dobutamine hydrochloride (DH)	YAP inhibition	Human osteoblastoma U2OS cells	Induced YAP phosphorylation at serine 127	[119]
Lysophosphatidic acid (LPA)	YAP/TAZ nuclear activation	iPSC-induced neuron	Increased YAP nuclear localization; inhibited neuron induction	[111]
		MSC derived from bovine bone marrow	Increased stress fiber; decreased contractility	[120]
Statin	Anti-atherosclerotic drug; YAP/TAZ inhibition	Human endothelial cells	Inhibited cell proliferation and anti-inflammatory effect	[86]
Sphingosine-1-phosphate (S1P)	YAP activation	HEK293A	Induced YAP nuclear localization	[121]
Verteporfin (VP)	YAP inhibition; blocked YAP-TEAD interaction	Human HSC-derived erythroid cells	Inhibited cell growth and differentiation	[122]

**Table 2:** Small-molecule-mediated YAP/TAZ activity through actin cytoskeleton.

Agents	Effectiveness	Cell Types	Treatment Results	References
Blebbistatin	Myosin inhibition	Mouse embryonic fibroblast NIH3T3	Reduced stress fibers F-actin and nuclear YAP	[116]
		Endothelial cells	Block shear stress-induced YAP translocation	[87]
Cytochalasin D	Actin inhibition; blocked actin polymerization, resulting in reduction of YAP and TEAD nuclear activity	Mouse embryonic fibroblast NIH3T3	Reduced stress fiber and nuclear YAP	[116]
		HEK293A	Blocked YAP dephosphorylation	[115]
		MSC derived from bovine bone marrow	Loss of stress fiber; decreased contractility	[120]
C3 exoenzyme	Rho-specific inhibition	HEK293A	Suppressed YAP/TAZ dephosphorylation	[115]
		Human ESCs	Massive cell death	[98]
		Human endothelial cells	Strongly regulated YAP/TAZ activity	[86]
CT04	Rho-GTPase inhibition	Human iPSC-derived hemogenic endothelial cells	Decreased Runx1/Cmyb expression	[88]
CN02, CN03 and CN04	Rho-GTPase activation	Human iPSC-derived hemogenic endothelial cells	Induced YAP signaling and promoted HSC formation	[88]
Latrunculin A	Actin inhibition; prevented the conversion of globular G-actin into filamentous F-actin and disrupted actin polymerization	MSCs	YAP/TAZ nuclear exclusion	[13]
		Mouse embryonic fibroblast NIH3T3	Reduced stress fiber and nuclear YAP	[116]
		Human PSCs	Decreased YAP/TEAD reporter activity	[70]
Latrunculin B	F-actin inhibition	HEK293A	Blocked YAP dephosphorylation and prevented YAP nuclear localization	[115]
		Nucleus pulposus cells of intervertebral disc	Reduced cell proliferation	[117]
Y-27632	ROCK inhibition	iPSC-induced neurons	Promoted YAP/TAZ cytoplasmic retention; increased the percentage of neuroepithelial progenitors	[111]
		Human periodontal ligament stem cells	Promoted cell proliferation, chemotaxis, and wound healing	[123]
		MSC derived from bovine bone marrow	Loss of stress fiber; decreased contractility	[120]
V14-Rho	RhoA activation	Human endothelial cells	Strongly regulated YAP/TAZ activity	[86]
